# Isatuximab in combination with cemiplimab in patients with relapsed/refractory multiple myeloma: A phase 1/2 study

**DOI:** 10.1002/cam4.5753

**Published:** 2023-03-03

**Authors:** Alexander Lesokhin, Richard LeBlanc, Meletios A. Dimopoulos, Marcelo Capra, Carmelo Carlo‐Stella, Lionel Karlin, Jean‐Francois Castilloux, Peter Forsberg, Gurdeep Parmar, Axel Tosikyan, Ludek Pour, Vincent Ribrag, Rossella Ribolla, Al‐Ola Abdallah, Nadia Le Roux, Liyan Dong, Helgi van de Velde, Laurent Mayrargue, Lucie Lépine, Sandrine Macé, Philippe Moreau

**Affiliations:** ^1^ Myeloma Service, Department of Medicine Memorial Sloan Kettering Cancer Center New York City New York USA; ^2^ Division of Hematology, Oncology and Transplantation, Department of Medicine Maisonneuve‐Rosemont Hospital, Université de Montréal Montréal Qubec Canada; ^3^ Department of Clinical Therapeutics National and Kapodistrian University of Athens Athens Greece; ^4^ Centro Integrado de Hematologia e Oncologia Hospital Mãe de Deus Porto Alegre Brazil; ^5^ Department of Biomedical Sciences Humanitas University Rozzano‐Milan Italy; ^6^ Department of Oncology and Hematology IRCCS – Humanitas Research Hospital Rozzano‐Milan Italy; ^7^ Department of Hematology Hôpital Lyon‐Sud, Hospices Civils de Lyon, Université Claude Bernard Lyon 1 Lyon France; ^8^ Centre Hospitalier Universitaire de Sherbrooke, Division of Hematology and Medical Oncology Université de Sherbrooke Sherbrooke Canada; ^9^ Department of Medicine, Division of Hematology University of Colorado School of Medicine Aurora Colorado USA; ^10^ Department of Haematology Wollongong Hospital Wollongong New South Wales Australia; ^11^ Hôpital du Sacré‐Coeur de Montréal Montréal Qubec Canada; ^12^ Department of Internal Medicine University Hospital Brno Brno Czech Republic; ^13^ Department of Hematology, Gustave Roussy Université Paris‐Saclay Villejuif France; ^14^ Department of Hematology ASST Spedali Civili di Brescia Brescia Italy; ^15^ Division of Hematological Malignancies and Cellular Therapeutics University of Kansas Lawrence Kansas USA; ^16^ Sanofi Research & Development on behalf of Altran Vitry‐sur‐Seine France; ^17^ Sanofi Beijing China; ^18^ Sanofi Cambridge Massachusetts USA; ^19^ Sanofi Research & Development Chilly‐Mazarin France; ^20^ Sanofi Clinical Pharmacokinetics on behalf of Excelya Chilly‐Mazarin France; ^21^ Sanofi Research & Development Vitry‐sur‐Seine France; ^22^ Hematology Department CHU Nantes Nantes France

## Abstract

**Background:**

Given the incurable nature of multiple myeloma (MM), efforts are made to improve the efficacy of anti‐CD38 monoclonal antibodies via combinations with other potentially synergistic therapies. This Phase 1/2 trial (NCT03194867) was designed to determine whether cemiplimab (anti‐PD‐1) enhances the anti‐myeloma activity of isatuximab (anti‐CD38) in patients with relapsed and refractory multiple myeloma (RRMM), to confirm the feasibility of the combination, determine its efficacy, and further evaluate its safety.

**Methods:**

Patients received isatuximab 10 mg/kg once weekly for 4 weeks followed by every 2 weeks (Isa), or isatuximab 10 mg/kg plus cemiplimab 250 mg every 2 (Isa + CemiQ2W) or every 4 weeks (Isa + CemiQ4W).

**Results:**

Overall, 106 patients with RRMM treated with a median of 4 prior lines were included; 25.5% had high‐risk cytogenetics, 63.2% were refractory to proteasome inhibitors and immunomodulatory agents, 26.4% were previously exposed to daratumumab, and 84.0% were refractory to their last treatment line. There were no major changes in the safety or pharmacokinetic profile of isatuximab with the addition of cemiplimab. As assessed by investigators, four patients (11.8%) in the Isa arm, nine patients (25.0%) in the Isa + CemiQ2W arm, and eight patients (22.2%) in the Isa + CemiQ4W arm were responders. Though response rates were numerically higher in cemiplimab‐containing arms, differences were not statistically significant and did not translate to improved progression‐free or overall survival after a median follow‐up of 9.99 months.

**Conclusion:**

Our results suggest a marginal benefit by adding cemiplimab to isatuximab, despite demonstration of target engagement, without additional observed safety issues.

## Graphical abstract

Preclinical data suggest that concurrent treatment of anti‐CD38 and anti‐PD‐1/PD‐L1 antibodies can substantially reduce primary tumor growth by reversing T‐cell exhaustion, and thus may synergize with anti‐PD‐1/PD‐L1 treatment. This Phase 1/2 trial was designed to determine whether cemiplimab (anti‐PD‐1) enhances the anti‐myeloma activity of isatuximab (anti‐CD38) in patients with relapsed and refractory multiple myeloma, to confirm the feasibility of the combination, determine its efficacy, and further evaluate its safety. We observed a marginal benefit by adding cemiplimab to isatuximab, with target engagement confirmed, and without additional safety issues observed.

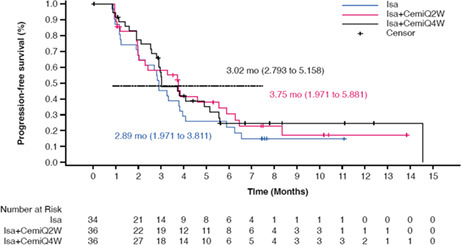

## 1 INTRODUCTION

Despite the advent of novel treatments over the past decade, multiple myeloma (MM) remains incurable and patients face multiple lines of therapy due to the relapsing and refractory nature of the disease. Antibodies that target CD38, a glycoprotein that is uniformly expressed on myeloma cells and plays important roles in immune‐system evasion,^1^ are increasingly incorporated into earlier lines of treatment for MM. Their efficacy for the treatment of relapsed and refractory MM (RRMM) is well established.^2^, ^3^, ^4^, ^5^, ^6^, ^7^

Isatuximab (Sarclisa®) is an immunoglobulin (Ig)G1 monoclonal antibody (mAb) that targets a specific epitope of CD38 and contributes to myeloma cell killing via multiple mechanisms of action.^8^, ^9^, ^10^ Based on the randomized, parallel‐group, Phase 3 ICARIA‐MM study (*n* = 307),^2^ isatuximab is approved in a number of countries in combination with pomalidomide and dexamethasone for the treatment of adult patients with RRMM who have received at least two prior lines of therapy, including lenalidomide and a proteasome inhibitor (PI). Based on the randomized, parallel‐group, Phase 3 IKEMA study (*n* = 302),^3^ to date, isatuximab in combination with carfilzomib and dexamethasone is approved in the United States, the European Union, and in Japan for patients who have received prior treatment.^11^, ^12^, ^13^

Based on studies in patients with RRMM showing clinical response with single‐agent isatuximab, which is enhanced in combination with immunomodulatory drugs (IMiDs), a rationale supports the combination of anti‐CD38 antibodies with other potentially synergistic immunomodulators.^14^, ^15^ Cemiplimab (Libtayo®) is a high‐affinity, hinge‐stabilized IgG4P fully human antibody that binds to programmed cell death protein 1 (PD‐1) and is designed to block PD‐1/programmed death ligand‐1 (PD‐L1) and −2 (PD‐L2)‐mediated T‐cell inhibition.^16^

Anti‐PD‐1 antibodies play a clear role in the treatment of various solid tumors, but their role in the treatment of MM has not been fully elucidated. As a single agent, the anti‐PD‐1 agent nivolumab showed minimal activity in a Phase 1 trial of 17 patients with RRMM, yielding a stable disease rate of 63%. Partial responses or better were not seen, with the exception of one complete response occurring after local radiation therapy.^17^ However, PD‐1/PD‐L1 interaction has been associated with the suppression of immune responses to myeloma cells,^18^ and preclinical studies indicated that isatuximab‐induced antibody‐dependent cellular cytotoxicity could be enhanced through the inhibition of PD‐1.^19^ Furthermore, a recent report demonstrated that combined PD‐L1 and CD38 inhibition improves antitumor immune responses in an animal model,^20^ suggesting that there may be benefit in combining these modalities.

The goal of this study was to determine whether cemiplimab may enhance the anti‐myeloma activity of isatuximab. This phase 1/2 trial (NCT03194867) is the first study of isatuximab plus cemiplimab designed to evaluate safety, tolerability, efficacy, and pharmacokinetics of the combination in RRMM. Due to previous Food and Drug Administration alerts on safety concerns with increasing risk of death regarding combinations of anti‐PD‐1/PD‐L1 agents with immunomodulatory agents (i.e., pomalidomide, lenalidomide) in the treatment of MM,^21^ the protocol for this study implemented careful safety monitoring by a Data Monitoring Committee.

## 2 METHODS

### 2.1 Study design and objectives

This was a Phase 1/2 randomized, parallel‐group, open‐label, multicenter study of isatuximab plus cemiplimab in patients with RRMM. Patients were enrolled at 29 sites in 10 countries (Australia, Brazil, Canada, Czech Republic, France, Greece, Hungary, Italy, Spain, and the United States). The study employed an independent Data Monitoring Committee to monitor the safety of patients enrolled in the study. The study was conducted in accordance with the Declaration of Helsinki and the International Conference on Harmonization Good Clinical Practice Guidelines, and individual independent ethics committees and institutional review boards approved the study protocol. All patients provided written informed consent prior to the conduct of any study‐related procedures.

The study comprised two phases. The primary objective of Phase 1 (safety run‐in) was to confirm the feasibility of isatuximab plus cemiplimab in patients with RRMM and to select the recommended Phase 2 dose (RP2D). The objective of the Phase 2 proof‐of‐concept study was to determine the efficacy of isatuximab plus cemiplimab for patients with RRMM and further evaluate the safety of the combination.

### 2.2 Participants

Eligible patients were ≥18 years of age, had a known diagnosis of MM with evidence of measurable disease, had received prior treatment with an IMiD and a PI, and had received ≥3 prior lines of therapy. Patients who previously received other anti‐CD38 mAbs were allowed if treatment was beyond 90 days from study entry, and if refractory, the last exposition was beyond 6 months. Exclusion criteria included prior exposure to isatuximab or any agent that blocks the PD‐1/PD‐L1 pathway; Eastern Cooperative Oncology Group performance status (ECOG PS) >2; and inadequate hematological, liver, or renal function.

### 2.3 Procedures

The Phase 1 portion of the study enrolled 3 eligible patients to receive the approved dose of isatuximab 10 mg/kg intravenously (IV) once weekly for 4 weeks followed by 10 mg/kg once every 2 weeks and cemiplimab 250 mg IV once every 2 weeks (Isa + CemiQ2W). In the case of dose‐limiting toxicities (DLTs), dose de‐escalation (Table 1) was planned using standard 3 + 3 methodology.

**TABLE 1:** Phase 1 treatment dose and schedule.

Dose level	Isatuximab	Cemiplimab
Dose level 1	10 mg/kg QWx4 > Q2W	250 mg Q2W
Dose level − 1	10 mg/kg QWx4 > Q2W	250 mg Q4W
Dose level − 2	10 mg/kg Q2W	250 mg Q4W

Abbreviations: QW, weekly; Q2W, every 2 weeks; Q4W, every 4 weeks.

The Phase 2 portion of the study randomly assigned eligible patients to one of three treatment arms in a 1:1:1 ratio using Interactive Response Technology. Patients in arm 1 (the control arm; Isa) received isatuximab 10 mg/kg once weekly for 4 weeks followed by once every 2 weeks. Patients in arm 2 received isatuximab 10 mg/kg once weekly for 4 weeks followed by once every 2 weeks plus cemiplimab 250 mg once every 2 weeks (Isa + CemiQ2W). Patients in arm 3 received Isa 10 mg/kg once weekly for 4 weeks followed by once every 2 weeks plus cemiplimab 250 mg once every 4 weeks (Isa + CemiQ4W). Alternative Phase 2 study designs were in place should DLTs during Phase 1 have changed the predicted Phase 2 treatment doses and schedules. All cycles were 28 days in length. Premedication with acetaminophen (650–1000 mg orally), ranitidine (50 mg IV), diphenhydramine (25–50 mg IV), and methylprednisolone (100 mg IV) was administered 30–60 minutes prior to isatuximab infusion. In both phases of the study, patients continued treatment until disease progression, unacceptable adverse events (AEs), consent withdrawal, or any other reason.

### 2.4 Outcomes

Primary endpoints for Phase 1 were safety and tolerability, which were assessed according to DLTs (at the end of Cycle 1) as well as AEs, serious AEs (SAEs), and laboratory abnormalities (graded by the National Cancer Institute Common Terminology Criteria for Adverse Events version 4.03). For Phase 2, the primary endpoint was overall response rate (ORR), defined as the proportion of patients with complete response (CR; including stringent CR [sCR]), very good partial response (VGPR), and partial response (PR), as assessed by investigators using the International Myeloma Working Group response criteria.^22^

Key secondary efficacy endpoints included clinical benefit rate (CBR), duration of response (DOR), time to response (TTR), progression‐free survival (PFS), overall survival (OS), pharmacokinetics (PK), and immunogenicity. Additional exploratory endpoints were minimal residual disease in patients achieving CR, and immune and tumor cell phenotyping.

Efficacy analyses for Phase 2 were performed for the intent‐to‐treat (ITT)/randomized population. ORR, CBR, PFS, and OS were separately assessed according to previous exposure to daratumumab. Analysis of exposure and safety parameters were performed for the all‐treated/safety population, which consisted of all patients who received at least one dose or a part of a dose of the study treatments during Phase 1 or 2 (patients were analyzed separately by phase). Pharmacokinetic analysis was performed on all patients from the all‐treated population with at least one measurable drug concentration post‐baseline.

### 2.5 Exploratory biomarkers and pharmacokinetic analysis

Cytogenetic analysis was performed for the all‐treated population who had one assessment on the biomarker of interest (collected from bone marrow and blood samples) unless otherwise specified.

Immunophenotyping analysis was performed for part of the safety population, consisting of all patients who received at least one dose or a part of a dose of the study treatments during Phase 2.

Blood samples were collected at selected time points over Cycle 1 to perform PK non‐compartmental analysis for Isa.

Additional details can be found in Methods S1.

### 2.6 Statistical analysis

Demographic and baseline characteristics were summarized using descriptive statistics. The primary analysis of ORR was performed at a cutoff date of 6 months after the last patient's first dose. ORR was summarized by treatment arms with descriptive statistics. A 95% two‐sided confidence interval was computed using the Clopper–Pearson method. Fisher's exact test was used to compare the ORR in the control arm versus each of the combination arms, using a one‐sided significance level of 0.10 with Hochberg adjustment. CBR and proportion of patients experiencing ≥VGPR as best overall response were summarized with descriptive statistics. DOR, TTR, PFS, and OS were analyzed using the Kaplan–Meier method.

## 3 RESULTS

### 3.1 Patients

A total of three patients were enrolled in Phase 1 at a dose of Isa + CemiQ2W. Two patients were male, all were between 63 and 68 years of age, and all had an ECOG PS of 0 or 1. Two patients discontinued study treatment due to progressive disease (PD); the other patient withdrew from the study after the first cycle (patient withdrawal). For Phase 2, 106 patients were randomized: 34 patients to isatuximab alone, 36 to Isa + CemiQ2W, and 36 to Isa + CemiQ4W. Overall, patient demographics and baseline disease characteristics were similar between all arms, with some differences observed mostly in the Isa + CemiQ2W arm with more prevalent high‐risk cytogenetics and International Staging System (ISS) stage III (Table 2).

**TABLE 2:** Summary of patient demographics and other baseline characteristics (ITT/randomized population).

	Isa (*n* = 34)	Isa + CemiQ2W (*n* = 36)	Isa + CemiQ4W (*n* = 36)	All (*N* = 106)
Age (years)				
Median (range)	68.0 (41–82)	64.0 (45–84)	66.5 (49–86)	66.0 (41–86)
Age group, *n* (%)				
<65 years	11 (32.4)	20 (55.6)	14 (38.9)	45 (42.5)
65–74 years	18 (52.9)	9 (25.0)	15 (41.7)	42 (39.6)
≥75 years	5 (14.7)	7 (19.4)	7 (19.4)	19 (17.9)
Race, *n* (%)				
Asian	0	0	1 (2.8)	1 (0.9)
Black or African American	2 (5.9)	3 (8.3)	3 (8.3)	8 (7.5)
White	29 (85.3)	30 (83.3)	29 (80.6)	88 (83.0)
Missing/Not reported	3 (8.8)	3 (8.3)	2 (5.6)	8 (7.5)
Unknown	0	0	1 (2.8)	1 (0.9)
ECOG PS, *n* (%)				
0	13 (38.2)	11 (30.6)	16 (44.4)	40 (37.7)
1	21 (61.8)	22 (61.1)	19 (52.8)	62 (58.5)
2	0	3 (8.3)	1 (2.8)	4 (3.8)
Cytogenetic risk status, *n* (%)				
High^a^	8 (23.5)	11 (30.6)	8 (22.2)	27 (25.5)
Standard	13 (38.2)	9 (25.0)	16 (44.4)	38 (35.8)
Unknown/missing	13 (38.2)	16 (44.4)	12 (33.3)	41 (38.7)
MM subtype at study entry, *n* (%)				
IgG	18 (52.9)	25 (69.4)	22 (61.1)	65 (61.3)
IgA	11 (32.4)	5 (13.9)	7 (19.4)	23 (21.7)
IgM	0	1 (2.8)	1 (2.8)	2 (1.9)
IgD	0	0	1 (2.8)	1 (0.9)
IgE	0	0	0	0
Kappa light chain only	2 (5.9)	2 (5.6)	4 (11.1)	8 (7.5)
Lambda light chain only	3 (8.8)	3 (8.3)	1 (2.8)	7 (6.6)
ISS stage at study entry, *n* (%)				
Stage I	7 (20.6)	15 (41.7)	14 (38.9)	36 (34.0)
Stage II	20 (58.8)	8 (22.2)	11 (30.6)	39 (36.8)
Stage III	7 (20.6)	13 (36.1)	8 (22.2)	28 (26.4)
Unknown	0	0	3 (8.3)	3 (2.8)
Creatinine clearance, *n* (%)				
<60 mL/min/1.73 m^2^	14 (41.2)	11 (30.6)	8 (22.2)	33 (31.1)
≥60 mL/min/1.73 m^2^	17 (50.0)	22 (61.1)	25 (69.4)	64 (60.4)
Missing	3 (8.8)	3 (8.3)	3 (8.3)	9 (8.5)
Bone marrow plasma cells at baseline, *n* (%)				
≥50%	10 (29.4)	11 (30.6)	8 (22.2)	29 (27.4)
Missing	1 (2.9)	1 (2.8)	0	2 (1.9)
Number of prior lines by patient				
Median (range)	4.0 (2.0–11.0)	4.0 (2.0–9.0)	4.0 (2.0–11.0)	4.0 (2.0–11.0)
2^b^	2 (5.9)	2 (5.6)	5 (13.9)	9 (8.5)
3	5 (14.7)	12 (33.3)	11 (30.6)	28 (26.4)
4	12 (35.3)	8 (22.2)	9 (25.0)	29 (27.4)
5	4 (11.8)	8 (22.2)	4 (11.1)	16 (15.1)
6	3 (8.8)	1 (2.8)	3 (8.3)	7 (6.6)
7	2 (5.9)	3 (8.3)	2 (5.6)	7 (6.6)
≥8	6 (17.6)	2 (5.6)	2 (5.6)	10 (9.4)
Main prior treatments, *n* (%)				
Alkylating agent	32 (94.1)	32 (88.9)	34 (94.4)	98 (92.5)
Immunomodulatory drug	34 (100)	36 (100)	36 (100)	106 (100)
Lenalidomide	29 (85.3)	36 (100)	28 (77.8)	93 (87.7)
Pomalidomide	20 (58.8)	22 (61.1)	14 (38.9)	56 (52.8)
Thalidomide	19 (55.9)	16 (44.4)	18 (50.0)	53 (50.0)
Proteasome inhibitor	34 (100)	35 (97.2)	36 (100)	105 (99.1)
Bortezomib	34 (100)	34 (94.4)	36 (100)	104 (98.1)
Carfilzomib	15 (44.1)	20 (55.6)	12 (33.3)	47 (44.3)
Ixazomib	4 (11.8)	4 (11.1)	2 (5.6)	10 (9.4)
Monoclonal antibodies	12 (35.3)	8 (22.2)	12 (33.3)	32 (30.2)
Daratumumab	11 (32.4)	6 (16.7)	11 (30.6)	28 (26.4)
Elotuzumab	1 (2.9)	3 (8.3)	5 (13.9)	9 (8.5)
Refractory status				
Refractory to IMiD	29 (85.3)	33 (91.7)	26 (72.2)	88 (83.0)
Refractory to PI	20 (58.8)	31 (86.1)	25 (69.4)	76 (71.7)
Refractory to IMiD and PI	19 (55.9)	29 (80.6)	19 (52.8)	67 (63.2)
Refractory to last regimen	27 (79.4)	33 (91.7)	29 (80.6)	89 (84.0)

Abbreviations: Cemi, cemiplimab; ECOG PS, Eastern Cooperative Oncology Group performance status; Ig, immunoglobulin; IMiD, immunomodulatory drug; Isa, isatuximab; ISS, International Staging System; ITT, intent‐to‐treat; MM, multiple myeloma; PI, proteasome inhibitor; Q2W, every 2 weeks; Q4W, every 4 weeks.

^a^ High‐risk defined as presence of del(17p) (10% cutoff), and/or t(4;14) (15% cutoff), and/or t(14;16) (15% cutoff).

^b^ Patients (*n* = 9) with <3 lines of therapy were included by mistake.

Patients were heavily pretreated. The median number of prior lines of therapy was 4 in each arm (up to 9 and 11), 37.7% of patients received ≥5 previous lines of therapy, and 84.0% were refractory to the last regimen. The vast majority was treated with alkylating agents, PIs, and IMiDs. The majority was double refractory to PIs and IMiDs. Overall, 28 patients (26.4%) had received previous daratumumab treatment, four of whom received daratumumab as the last line of therapy. The median time from last dose of daratumumab to first dose of isatuximab was 9 (range, 2.1–22.1) months; some patients received the first dose of isatuximab after a shorter washout period than recommended.

At the data cutoff date (October 9, 2019), 14 patients remained on treatment, including five patients each in the Isa and Isa + CemiQ2W arm and four patients in the Isa + CemiQ4W arm. Across all arms, the most common reason for definitive treatment discontinuation was PD (Isa, 82.4%; Isa + CemiQ2W, 72.2%; Isa + CemiQ4W, 66.7%) (Table S1).

### 3.2 Efficacy

The median follow‐up duration was 9.99 months (95% CI, 8.542–10.875) across all study arms (*N* = 106). A response was seen in four patients (11.8%) in the Isa arm, nine patients (25.0%) in the Isa + CemiQ2W arm, and eight patients (22.2%) in the Isa + CemiQ4W arm (Table 3). Differences in ORR between arms were not statistically different. The differences between the Isa arm and the Isa + CemiQ2W arm (*p =* 0.1321; one‐sided Fisher's exact test) and between the Isa arm and the Isa + CemiQ4W (*p =* 0.2003; one‐sided Fisher's exact test) were not statistically significant. As the *p* values were greater than 0.1, the null hypothesis failed to be rejected for both combination arms. No CR was observed in any treatment arm and a similar number of patients in each arm achieved VGPR: two patients (5.9%) in the Isa arm, three patients (8.3%) in the Isa + CemiQ2W arm, and two patients (5.6%) in the Isa + CemiQ4W arm.

**TABLE 3:** Response rates (ITT/randomized population).

	Isa (*n* = 34)	Isa + CemiQ2W (*n* = 36)	Isa + CemiQ4W (*n* = 36)
Best overall response, *n* (%)			
Stringent complete response	0	0	0
Complete response	0	0	0
Very good partial response	2 (5.9)	3 (8.3)	2 (5.6)
Partial response	2 (5.9)	6 (16.7)	6 (16.7)
Minimal response	4 (11.8)	4 (11.1)	6 (16.7)
Stable disease	14 (41.2)	12 (33.3)	16 (44.4)
Progressive disease	7 (20.6)	6 (16.7)	4 (11.1)
Unconfirmed progressive disease	1 (2.9)	2 (5.6)	1 (2.8)
Not evaluable/Not assessed	4 (11.8)	3 (8.3)	1 (2.8)
Overall response			
Responders (sCR, CR, VGPR, or PR)	4 (11.8)	9 (25.0)	8 (22.2)
95% CI^a^	3.30–27.45	12.12–42.20	10.12–39.15
Fisher's exact test *p* value^b^			
Isa + CemiQ2W vs Isa		0.1321	
Isa + CemiQ4W vs Isa			0.2003
Clinical benefit			
Responders (MR or better)	8 (23.5)	13 (36.1)	14 (38.9)
95% CI^a^	10.75–41.17	20.82–53.78	23.14–56.54

Abbreviations: Cemi, cemiplimab; CI, confidence interval; CR, complete response; Isa, isatuximab; ITT, intent‐to‐treat; MR, minimal response; PR, partial response; Q2W, every 2 weeks; Q4W, every 4 weeks; sCR, stringent complete response; VGPR, very good partial response.

^a^ Estimated using Clopper–Pearson method.

^b^ One‐sided significance level of 0.1.

No responses were observed in 28 (26%) patients previously treated with daratumumab (anti‐CD38 monoclonal antibody targeting a different CD38 epitope than Isa) across the three arms. The ORR among patients not previously treated with daratumumab was 17.4% in the Isa arm, 30.0% in the Isa + CemiQ2W arm, and 32.0% in the Isa + CemiQ4W arm (Table S2). The CBR was higher in cemiplimab‐treated arms versus the Isa arm among patients not previously treated with daratumumab.

**FIGURE 1:**
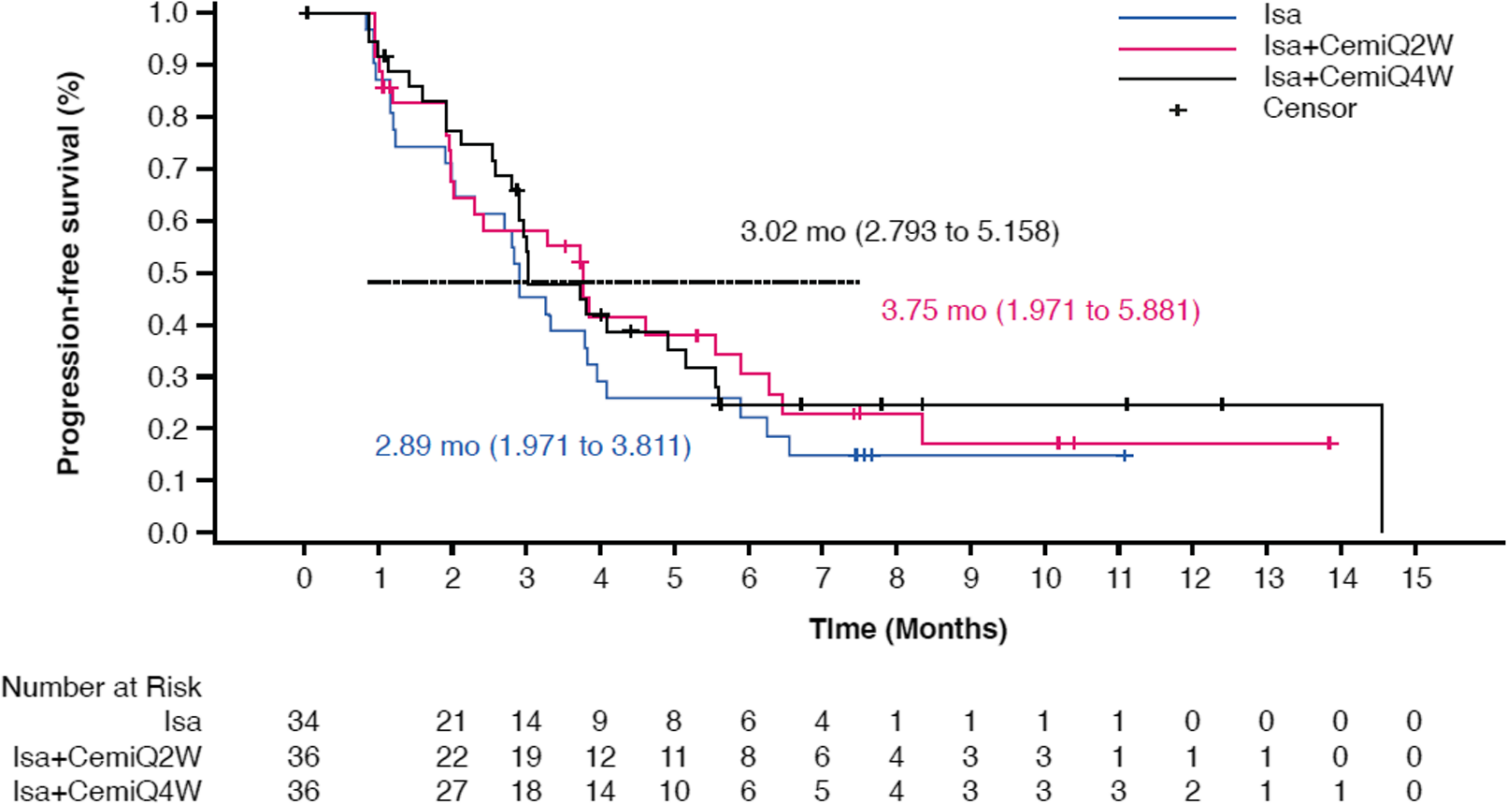
Progression‐free survival across treatment arms (ITT/Randomized group). Cemi, cemiplimab; Isa, isatuximab; Q2W, every 2 weeks; Q4W, every 4 weeks.

Median PFS (95% CI) was similar between arms, at 2.89 months (1.97–3.81) for the Isa arm, 3.75 months (1.97–5.88) for the Isa + CemiQ2W arm, and 3.02 months (2.79–5.16) for the Isa + CemiQ4W arm (Figure 1).

Across arms, median PFS was consistently higher among patients without versus with previous daratumumab exposure. For patients not previously treated with daratumumab, median PFS (95% CI) was 3.32 months (2.00–5.88) for the Isa arm, 3.75 months (2.00–6.28) for the Isa + CemiQ2W arm, and 4.07 months (2.89–14.55) for the Isa + CemiQ4W arm. For patients previously treated with daratumumab, median PFS (95% CI) was 2.14 months (0.92–3.25) for the Isa arm, 1.97 months (0.95–not reached [NR]) for the Isa + CemiQ2W arm, and 2.87 months (1.12–3.02) for the Isa + CemiQ4W arm.

**FIGURE 2:**
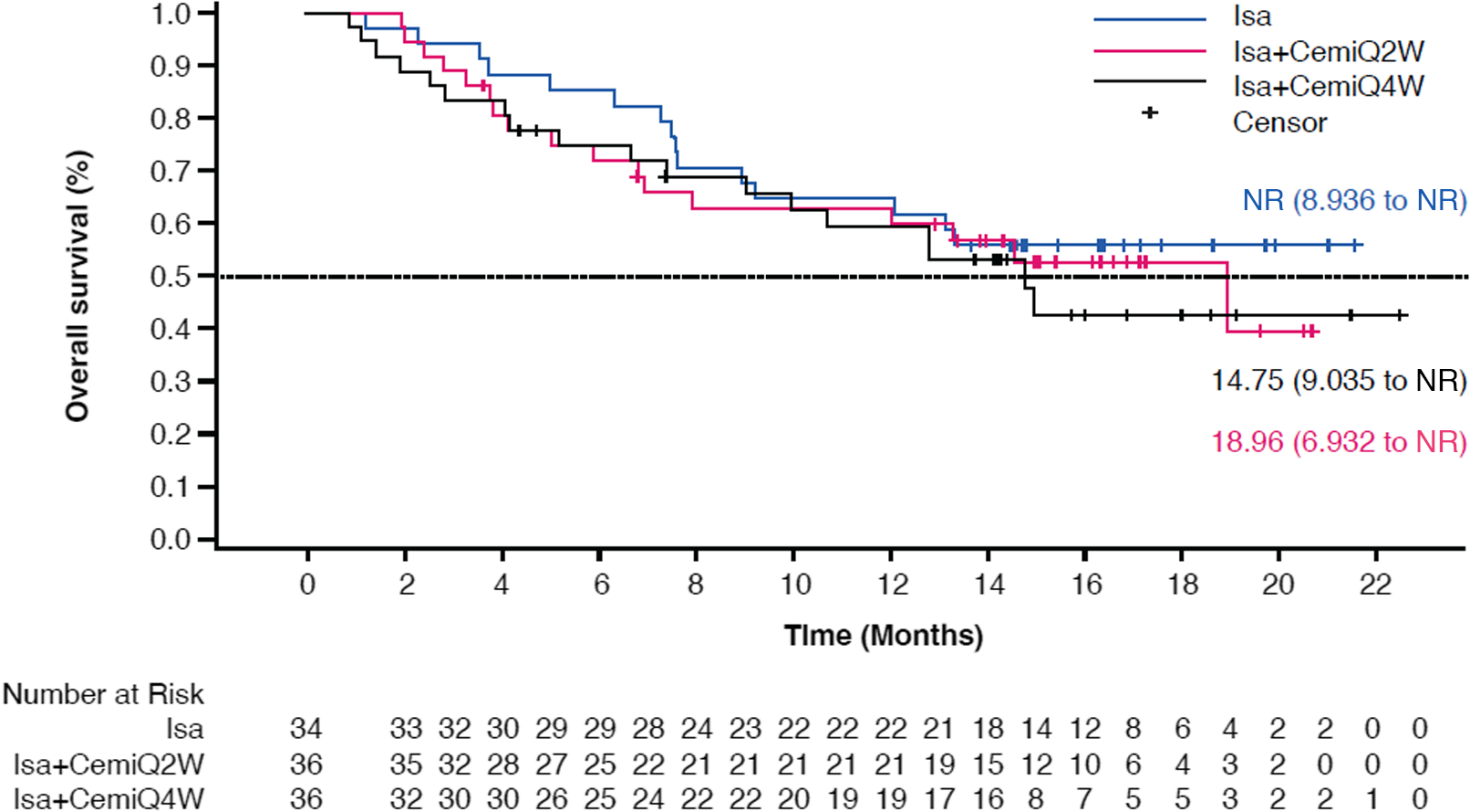
Overall survival across treatment arms (ITT/randomized population). Cemi, cemiplimab; Isa, isatuximab; NR, not reached; Q2W, every 2 weeks; Q4W, every 4 weeks.

At the time of updated OS analysis (cutoff date: April 9, 2020; median follow‐up: Isa—16.79 months; Isa + CemiQ2W—16.16 months; Isa + CemiQ4W—15.74 months), OS was similar among arms, with largely overlapping OS curves. Median OS (95% CI) was NR for the Isa arm (8.936–NR), 18.96 (6.932–NR) months for the Isa + CemiQ2W arm, and 14.75 (9.04–NR) months for patients in the Isa + CemiQ4W arm (Figure 2). For the 28 (26%) daratumumab‐exposed patients, OS was also similar between arms.

### 3.3 Safety

No DLTs were observed during the Phase 1 portion of the study. The all‐treated/safety population comprised 33 patients treated with Isa alone, 37 patients treated with Isa + CemiQ2W, and 35 patients treated with Isa + CemiQ4W (two patients were randomized to a treatment arm but received another one, so they were kept in the randomized arm). The median number of cycles (range) started by patients was 4 (1–12) in the Isa arm, 3 (1–14) in the Isa + CemiQ2W arm, and 4 (1–15) in the Isa + CemiQ4W arm. At the time of data cutoff, the median duration of exposure (range) to isatuximab was 14.43 weeks (2.0–48.0) in the Isa arm, 12.57 weeks (1.0–59.4) in the Isa + CemiQ2W arm, and 14.29 weeks (3.0–62.9) in the Isa + CemiQ4W arm. The median duration of exposure (range) to cemiplimab was 12.57 weeks (2.0–59.4) in the Isa + CemiQ2W arm and 16.29 weeks (4.0–62.9) in the Isa + CemiQ4W arm.

At least 1 treatment‐emergent adverse event (TEAE) was reported in 100% of the Isa arm, 97.3% of the Isa + CemiQ2W arm, and 94.3% of the Isa + CemiQ4W arm (Table 4). Grade ≥3 TEAEs were reported in 51.5%, 54.1%, and 65.7% of patients in each of the arms, respectively. The number of patients with treatment‐related TEAEs of any grade was similar among arms. The number of patients with Grade ≥3 treatment‐related TEAEs was greatest in the Isa + CemiQ4W arm (10 [28.6%]). TEAEs reported in at least 15% of patients are shown in Table 5. One patient in the Isa + CemiQ4W arm developed a secondary malignancy (colon neoplasm, non‐fatal). No immune‐related TEAEs were reported.

**TABLE 4:** Overview of TEAEs (All‐treated/safety population).

	Isa (*n* = 33)	Isa + CemiQ2W (*n* = 37)	Isa + CemiQ4W (*n* = 35)
Patients with any TEAE	33 (100)	36 (97.3)	33 (94.3)
Patients with any Grade ≥3 TEAE	17 (51.5)	20 (54.1)	23 (65.7)
Patients with any Grade 3–4 TEAE	17 (51.5)	18 (48.6)	21 (60.0)
Patients with any Grade 5 TEAE^a^	1 (3.0)	2 (5.4)	6 (17.1)
Patients with any treatment‐emergent SAE	17 (51.5)	17 (45.9)	21 (60.0)
Patients with any TEAE leading to definitive treatment discontinuation^b^	0	1 (2.7)	7 (20.0)
Patients with any IR of Grade ≥2	16 (48.5)	15 (40.5)	14 (40.0)
Patients with any treatment‐related TEAE^c^ (any grade)	24 (72.7)	24 (64.9)	25 (71.4)
Patients with any Grade ≥3 treatment‐related TEAE	2 (6.1)	6 (16.2)	10 (28.6)
Patients with any serious treatment‐related TEAE	2 (6.1)	4 (10.8)	9 (25.7)

Abbreviations: Cemi, cemiplimab; IR, infusion reaction; Isa, isatuximab; Q2W, every 2 weeks; Q4W, every 4 weeks; SAE, serious adverse event; TEAE, treatment‐emergent adverse event.

^a^ Grade 5 TEAEs during the on‐treatment period included: Isa−disease progression (*n* = 1); Isa + CemiQ2W–disease progression (*n* = 1), multiple organ dysfunction syndrome (*n* = 1); Isa + CemiQ4W–pulmonary sepsis (*n* = 1; related to treatment); upper respiratory tract infection (*n* = 1); euthanasia (*n* = 1); acute kidney injury/disease progression (*n* = 1); disease progression (*n* = 1); septic shock (*n* = 1).

^b^ TEAEs leading to discontinuation included: Isa + CemiQ2W–myalgia (*n* = 1); Isa + CemiQ4W–encephalomyelitis (*n* = 1); pulmonary sepsis (*n* = 1); colon neoplasm (*n* = 1); upper respiratory tract infection (*n* = 1); infusion‐related reaction (*n* = 1); lower respiratory tract infection/septic shock (*n* = 1); peripheral sensory neuropathy (*n* = 1).

^c^ Treatment‐related TEAEs are TEAEs related to at least one drug of the combination.

**TABLE 5:** Summary of TEAEs occurring in at least 15% of patients (All‐treated/safety population).

	Isa (*n* = 33)		Isa + CemiQ2W (*n* = 37)		Isa + CemiQ4W (*n* = 35)	
	All grades	Grade ≥3	All grades	Grade ≥3	All grades	Grade ≥3
Infections and infestations	19 (57.6)	8 (24.2)	20 (54.1)	9 (24.3)	23 (65.7)	9 (25.7)
Upper respiratory tract infection	4 (12.1)	1 (3.0)	4 (10.8)	0	9 (25.7)	2 (5.7)
Gastrointestinal disorders	11 (33.3)	3 (9.1)	16 (43.2)	2 (5.4)	13 (37.1)	4 (11.4)
Diarrhea	6 (18.2)	1 (3.0)	7 (18.9)	0	7 (20.0)	2 (5.7)
Nausea	5 (15.2)	0	6 (16.2)	1 (2.7)	5 (14.3)	0
Musculoskeletal and connective tissue disorders	17 (51.5)	2 (6.1)	15 (40.5)	1 (2.7)	10 (28.6)	5 (14.3)
Back pain	4 (12.1)	1 (3.0)	6 (16.2)	0	4 (11.4)	2 (5.7)
General disorders and administration site conditions	15 (45.5)	2 (6.1)	18 (48.6)	4 (10.8)	10 (28.6)	3 (8.6)
Fatigue	3 (9.1)	0	8 (21.6)	0	5 (14.3)	1 (2.9)
Pyrexia	4 (12.1)	0	8 (21.6)	1 (2.7)	2 (5.7)	1 (2.9)
Injury, poisoning, and procedural complications	20 (60.6)	0	15 (40.5)	0	17 (48.6)	0
Infusion‐related reaction	18 (54.5)	0	15 (40.5)	0	16 (45.7)	0^a^

Abbreviations: Cemi, cemiplimab; Isa, isatuximab; Q2W, every 2 weeks; Q4W, every 4 weeks.

^a^ One patient in the Isa + CemiQ4W arm experienced a Grade 3 infusion reaction leading to hospitalization and investigational medicine product discontinuation, which was mistakenly reported as a Grade 2 infusion reaction.

Overall, nine patients died during the on‐treatment period. The main reason for death was due to progressive disease, including one patient in the Isa arm, two patients in the Isa + CemiQ2W arm, and two patients in the Isa + CemiQ4W arm. Overall, four patients in the Isa + CemiQ4W arm experienced a fatal TEAE during the on‐treatment period. Among them, one patient had pulmonary sepsis related to study treatment and three patients had fatal TEAEs unrelated to study treatment, including upper respiratory tract infection, euthanasia (the patient asked for euthanasia due to uncontrollable pain in the context of progressive disease), and septic shock. There was one TEAE‐related death during the post‐treatment period.

Infusion reactions (IRs) of any grade were experienced by 18 patients (54.5%) of patients in the Isa arm, 15 patients (40.5%) in the Isa + CemiQ2W arm, and 16 patients (45.7%) in the Isa + CemiQ4W arm. There were 2 Grade 1 IRs related to cemiplimab. Details of IRs are provided in Table S3. A trend was observed of lower incidence of IR in patients receiving isatuximab with cemiplimab. Onset of all IRs resulted from the first infusion.

### 3.4 Immunophenotyping analysis

Median relative change from baseline to Cycle 3, Day 1 (C3D1) of a panel of immune cells according to treatment arms (pool of Isa + Cemi arms vs Isa arm) was calculated using patients in the safety population (Figure S1). Baseline comparisons of clonal plasma cells and a panel of immune cells were also conducted between the daratumumab‐pretreated group and the daratumumab‐naïve group (Figure S2). Additional details are in Material S1.

### 3.5 Pharmacokinetics

Isa mean PK parameters for patients treated with single‐agent Isa, with the combination of Isa plus cemiplimab (any schedule), and overall are provided in Table S4. Concomitant administration of cemiplimab did not appear to alter Isa exposure (*C*_max_, AUC_1week_).

## 4 DISCUSSION

This Phase 1/2 study was designed to evaluate the safety, preliminary efficacy, and PK of the combination of Isa and cemiplimab in patients with RRMM, and is the largest randomized trial investigating the combination of anti‐CD38 and anti‐PD‐1 agents to date. The study population was representative of the global population with RRMM, including important subgroups of patients with poor prognosis characteristics (ISS Stage III [26.4%], renal function impairment with creatinine clearance <60 mL/min [31.1%], high‐risk cytogenetics [25.5%], and bone marrow plasma cells at baseline ≥50% [27.4%]). Patients were heavily pretreated, with a median (range) of 4 (2–11) prior lines of therapy. In the total randomized population, 37.7% of patients received ≥5 previous lines of therapy and 84.0% were refractory to the last regimen. Most patients were refractory to PIs and IMiDs. Overall, 26.4% of patients had received prior daratumumab, including 3.8% who received daratumumab within the last line of therapy.

No new safety signals were observed with Isa plus cemiplimab, which is consistent with other safety experiences with this combination^23^ and with daratumumab plus atezolizumab^24^ in solid malignancies. Compared with the risk of death observed in the current study, interim results of studies combining the PD‐1 inhibitor pembrolizumab with either pomalidomide or lenalidomide for patients with RRMM revealed an increased risk of death when pembrolizumab was added to the immunomodulatory drug.^21^ In KEYNOTE‐183 (pembrolizumab with pomalidomide), non‐disease progression causes of death were identified in the pembrolizumab arm, including myocarditis, Stevens‐Johnson syndrome, myocardial infarction, and pericardial hemorrhage.^25^ In KEYNOTE‐185 (pembrolizumab with lenalidomide), causes of death included intestinal ischemia, cardio‐respiratory arrest, and suicide.^26^

In the present study, we did not observe death due to cardiovascular or ischemic reasons, and there were few infections/sepsis events reported. Concomitant administration of cemiplimab did not appear to alter Isa exposure. With further follow‐up, the OS curves were overlapping when comparing Isa alone with Isa plus cemiplimab. This may be due to the different mechanism of action of isatuximab versus immunomodulatory drugs.

As assessed by Investigators, four patients (11.8%) in the Isa arm, nine patients (25.0%) in the Isa + CemiQ2W arm, and eight patients (22.2%) in the Isa + CemiQ4W arm were responders. Though response rates were numerically higher among cemiplimab‐containing arms, differences were not statistically significant and did not translate to improved PFS or OS. These findings of a modest improvement in ORR and lack of translation to PFS benefit are in contrast to what was shown previously with isatuximab in combination with pomalidomide and dexamethasone (ICARIA) and with carfilzomib and dexamethasone (IKEMA), which demonstrated significant benefits in PFS and response or depth of response.^2^, ^3^ However, these findings are consistent with a previous trial showing little clinical benefit achieved with a different PD‐1 inhibitor, nivolumab, among patients with RRMM.^17^ Interestingly, in the nivolumab study, immune‐mediated adverse events were reported in 28 (34%) of patients compared with none reported in the current study. As such, preclinical data^19^ suggesting that PD‐1 inhibition may increase the myeloma cell killing conferred by CD38 inhibition has yet to translate into meaningful benefit in clinical trials. Interestingly, results for ORR (11.8%), median PFS (2.89 months), and median OS (NR) in the Isa‐only arm were lower than those from other recent Isa studies.^14^, ^27^

In the present study, an Isa dose of 10 mg/kg was chosen to allow for the assessment of cemiplimab contribution. This dose was also used for the Isa‐only arm monotherapy studies. The lower response rates in Isa‐only arm of this study could be due to the use of the 10 mg/kg Isa dose versus the now‐recommended single‐agent^28^ and Japan‐approved dose of 20 mg/kg.^29^ Had the higher 20 mg/kg dose been used in the Isa‐only arm of the present study, it is possible that response rates would have been even more similar across study arms. The present study included daratumumab‐pretreated patients (~26% of the overall randomized population), whereas other monotherapy studies^27^, ^29^ excluded patients with prior anti‐CD38 therapy. There was some imbalance in prior daratumumab exposure, with higher rates in the monotherapy group. Additionally, some patients with prior daratumumab exposure were treated after a washout period of less than the recommended 6 months. In a recent study by Dimopoulos et al.^27^ Isa combined with dexamethasone contributed to increased response rates and survival outcomes compared with Isa alone. The ORR was 23.9% (isatuximab) versus 43.6% (isatuximab‐dexamethasone), with median PFS of 4.9 (isatuximab) versus 10.2 (isatuximab‐dexamethasone) months and median OS of 18.9 (isatuximab) versus 17.3 (isatuximab‐dexamethasone) months.

Whether benefit can be gained from an alternate anti‐CD38 agent (or even retreatment with the same anti‐CD38 agent) and the appropriate timing of reintroduction of CD38 inhibition remain important clinical questions, particularly as anti‐CD38 therapy moves into earlier lines of treatment. It is becoming increasingly clear that single‐agent anti‐CD38 treatment with isatuximab or daratumumab is insufficiently active in patients with prior anti‐CD38 exposure. In the present study, 28 of 106 (26.4%) patients in the overall randomized population had previously received daratumumab treatment, of whom four had received daratumumab as the last line of therapy, with no objective responses observed. In ICARIA‐MM, 22 of 92 (23.9%) patients treated with further anti‐myeloma therapy in the Isa‐Pd arm went on to receive daratumumab in any subsequent line of therapy, of whom seven received monotherapy (+/−steroids). The ORR for daratumumab as further anti‐myeloma treatment (in monotherapy) was lower after Isa‐Pd than after Pd treatment (14% vs 38%, respectively).^30^ In a recent Phase 1/2 clinical study of Isa monotherapy in daratumumab‐refractory patients, similarly, no objective responses were observed; however, the disease control rate (defined as ≥MR or stable disease for ≥8 weeks) was higher in patients who received their first dose of Isa monotherapy longer after their last daratumumab dose (60.0%, 58.3%, and 28.6% in those with ≥12, ≥6, and <3 months of daratumumab washout, respectively).^31^ In a UK‐wide, real‐world outcomes study of five patients receiving isatuximab–pomalidomide–dexamethasone, one patient previously received daratumumab monotherapy and four patients previously received daratumumab–bortezomib–dexamethasone. Following a median of 2 cycles of isatuximab–pomalidomide–dexamethasone, three patients had progressive disease, one patient had minor response‐stable disease, and one patient had a partial response.^32^ Results from the present study add to the available data on the appropriate use and timing of subsequent anti‐CD38 therapy, but this important clinical question should be addressed further in prospective crossover or retreatment studies such as NCT03871829.

Treatment with isatuximab with or without cemiplimab induced the expected pharmacodynamic effects, consistent with the mechanism of action of each drug as shown in previous studies.^23^ A decrease in the percentage of cells expressing CD38 on isatuximab treatment and PD‐1 on cemiplimab treatment at Cycle 3 was observed, as well as a decrease of CD38 expression on plasma cells. Target engagement was demonstrated, and as such, the low proportion of positive clinical outcomes did not appear to be attributable to failure of the drugs to adequately engage the relevant proteins.

Target engagement was shown to be similar in the population pretreated with or naïve to daratumumab, so there was no lack of target engagement that could explain the lack of response in the daratumumab‐pretreated population.

The baseline immune microenvironment in daratumumab‐pretreated patients appears to be consistent with what is known to happen after treatment with an anti‐CD38 molecule, that is, an increase in activated PD‐1+ CD8 T cells and proliferative Ki67+ CD8 T cells as well as a decrease in CD38+ CD8 T cells. The other baseline immune parameters, including natural killer cell counts, were similar between groups and did not explain the lack of response in daratumumab‐pretreated patients.

The current study has several limitations and strengths. Limitations of the current study include the small number of patients enrolled in each study arm, the heavily pretreated population, and the short washout period after prior exposure to anti‐CD38 agents. Strengths of the current study include biomarker data and continued demonstration of a manageable safety profile with the combination of isatuximab plus cemiplimab and of no effect of cemiplimab on isatuximab PK, which confirms experiences from other Isa plus cemiplimab studies.^23^

In conclusion, this study evaluating isatuximab in combination with cemiplimab in patients with RRMM indicates a marginal benefit on clinical outcomes with the addition of cemiplimab to isatuximab. The numerical increase in ORR seen with the addition of cemiplimab was not statistically significant and did not translate into an improvement in PFS or OS. No major changes in the safety or PK profile were observed with the addition of cemiplimab.

## AUTHOR CONTRIBUTIONS

**Alexander Lesokhin:** Conceptualization (equal); data curation (equal); formal analysis (equal); writing – original draft (equal); writing – review and editing (equal). **Richard LeBlanc:** Data curation (equal); formal analysis (equal); writing – review and editing (equal). **MA Dimopoulos:** Conceptualization (equal); data curation (equal); formal analysis (equal); writing – review and editing (equal). **Marcelo Capra:** Conceptualization (equal); data curation (equal); formal analysis (equal); writing – review and editing (equal). **Carmelo Carlo‐Stella:** Conceptualization (equal); data curation (equal); formal analysis (equal); writing – review and editing (equal). **lionel Karlin:** Data curation (equal); formal analysis (equal); writing – review and editing (equal). **Jean‐Francois Castilloux:** Data curation (equal); writing – review and editing (equal). **Peter Forsberg:** Conceptualization (equal); data curation (equal); formal analysis (equal); writing – review and editing (equal). **Gurdeep Parmar:** Conceptualization (equal); formal analysis (equal); writing – review and editing (equal). **Axel Tosikyan:** Formal analysis (equal); writing – review and editing (equal). **Ludek Pour:** Data curation (equal); formal analysis (equal); writing – review and editing (equal). **Vincent Ribrag:** Data curation (equal); formal analysis (equal); writing – review and editing (equal). **Rossella Ribolla:** Data curation (equal); writing – review and editing (equal). **Al‐Ola Abdallah:** Formal analysis (equal); writing – review and editing (equal). **Nadia Le Roux:** Conceptualization (equal); formal analysis (equal); writing – original draft (equal); writing – review and editing (equal). **Liyan Dong:** Formal analysis (equal); writing – original draft (equal); writing – review and editing (equal). **Helgi van de Velde:** Conceptualization (equal); formal analysis (equal); writing – original draft (equal); writing – review and editing (equal). **Laurent Mayrargue:** Conceptualization (equal); formal analysis (equal); writing – original draft (equal); writing – review and editing (equal). **Lucie Lépine:** Conceptualization (equal); data curation (equal); formal analysis (equal); writing – original draft (equal); writing – review and editing (equal). **Sandrine Macé:** Conceptualization (equal); formal analysis (equal); writing – original draft (equal); writing – review and editing (equal). **Philippe MOREAU:** Conceptualization (equal); data curation (equal); formal analysis (equal); writing – original draft (equal); writing – review and editing (equal).

## FUNDING INFORMATION

This study was sponsored by Sanofi.

## CONFLICT OF INTEREST STATEMENT

A.L.: Honoraria—Sanofi, Trillium Therapeutics, Pfizer, Janssen, Iteos; Research Funding—Bristol Myers Squibb, Pfizer, Janssen; Patent US20150037346A1. R.L.: Advisory Role—Janssen, Bristol Myers Squibb, Amgen, Sanofi, FORUS Therapeutics. M.A.D.: Advisory Role—Amgen, Beigene, Bristol Myers Squibb, Janssen, Takeda. M.C.: Honoraria—Bristol Myers Squibb, Janssen, Sanofi; Advisory Role—Janssen, Sanofi. C.C.‐S.: Honoraria—ADC Therapeutics, AstraZeneca, Bristol Myers Squibb, Incyte, Janssen, Takeda; Advisory Role—ADC Therapeutics, Bristol Myers Squibb, Celgene, Karyopharm Therapeutics, Novartis, Roche, Sanofi. L.K.: Honoraria—AbbVie, Amgen, Celgene, Janssen, Sanofi, Takeda; Advisory Role—Amgen, Celgene, GSK, Janssen, Takeda. J.‐F.C.: Nothing to disclose. P.F.: Honoraria—Celgene; Advisory Role—Bristol Myers Squibb, GlaxoSmithKline, Sanofi. G.P.: Research Funding—Bristol Myers Squibb; Advisory Role—Bristol My ers Squibb, Janssen, Sanofi. A.T.: Honoraria—Astellas, Bayer. L.P.: Nothing to disclose. V.R.: Advisory Role—AstraZeneca, Bristol Myers Squibb, EPZ, Incyte, MSD, nanostring, Pharmamar, Servier. R.R.: Nothing to disclose. A.‐O.A.: Nothing to disclose. N.L.R. is employed by Altran on behalf of Sanofi and may hold stock and/or stock options in the company. L.D., H.v.d.V., and S.M. are employed by Sanofi and may hold stock and/or stock options in the company. L.M. and L.L. are employed by Excelya on behalf of Sanofi and may hold stock and/or stock options in the company. P.M.: Honoraria—AbbVie, Amgen, Celgene, GlaxoSmithKline, Janssen, Sanofi; Advisory Role—AbbVie, Amgen, Celgene, GlaxoSmithKline, Janssen, Sanofi.

## Supporting information


Data S1.

## Data Availability

Qualified researchers can request access to patient‐level data and related study documents including the clinical study report, study protocol with any amendments, blank case report forms, statistical analysis plan, and dataset specifications. Patient‐level data will be anonymized, and study documents will be redacted to protect the privacy of trial participants. Further details on Sanofi's data‐sharing criteria, eligible studies, and process for requesting access are at: https://www.vivli.org.

## References

[1] van de Donk N , Richardson PG , Malavasi F . CD38 antibodies in multiple myeloma: back to the future. Blood. 2018;131(1):13‐29. 29118010 10.1182/blood-2017-06-740944

[2] Attal M , Richardson PG , Rajkumar SV , et al. Isatuximab plus pomalidomide and low‐dose dexamethasone versus pomalidomide and low‐dose dexamethasone in patients with relapsed and refractory multiple myeloma (ICARIA‐MM): a randomised, multicentre, open‐label, phase 3 study. Lancet. 2019;394(10214):2096‐2107. 31735560 10.1016/S0140-6736(19)32556-5

[3] Moreau P , Dimopoulos MA , Mikhael J , et al. Isatuximab, carfilzomib, and dexamethasone in relapsed multiple myeloma (IKEMA): a multicentre, open‐label, randomised phase 3 trial. Lancet. 2021;397(10292):2361‐2371. 34097854 10.1016/S0140-6736(21)00592-4

[4] Dimopoulos M , Quach H , Mateos MV , et al. Carfilzomib, dexamethasone, and daratumumab versus carfilzomib and dexamethasone for patients with relapsed or refractory multiple myeloma (CANDOR): results from a randomised, multicentre, open‐label, phase 3 study. Lancet. 2020;396(10245):186‐197. 32682484 10.1016/S0140-6736(20)30734-0

[5] Dimopoulos MA , Oriol A , Nahi H , et al. Daratumumab, lenalidomide, and dexamethasone for multiple myeloma. N Engl J Med. 2016;375(14):1319‐1331. 27705267 10.1056/NEJMoa1607751

[6] Palumbo A , Chanan‐Khan A , Weisel K , et al. Daratumumab, bortezomib, and dexamethasone for multiple myeloma. N Engl J Med. 2016;375(8):754‐766. 27557302 10.1056/NEJMoa1606038

[7] Dimopoulos MA , Terpos E , Boccadoro M , et al. Daratumumab plus pomalidomide and dexamethasone versus pomalidomide and dexamethasone alone in previously treated multiple myeloma (APOLLO): an open‐label, randomised, phase 3 trial. Lancet Oncol. 2021;22(6):801‐812. 34087126 10.1016/S1470-2045(21)00128-5

[8] Deckert J , Wetzel MC , Bartle LM , et al. SAR650984, a novel humanized CD38‐targeting antibody, demonstrates potent antitumor activity in models of multiple myeloma and other CD38+ hematologic malignancies. Clin Cancer Res. 2014;20(17):4574‐4583. 24987056 10.1158/1078-0432.CCR-14-0695

[9] Jiang H , Acharya C , An G , et al. SAR650984 directly induces multiple myeloma cell death via lysosomal‐associated and apoptotic pathways, which is further enhanced by pomalidomide. Leukemia. 2016;30(2):399‐408. 26338273 10.1038/leu.2015.240

[10] Moreno L , Perez C , Zabaleta A , et al. The mechanism of action of the anti‐CD38 monoclonal antibody isatuximab in multiple myeloma. Clin Cancer Res. 2019;25(10):3176‐3187. 30692097 10.1158/1078-0432.CCR-18-1597

[11] Sarclisa® (isatuximab‐irfc). Sanofi‐Aventis U.S. LLC; 2021.

[12] Sarclisa® (isatuximab). Sanofi Co., Ltd.; 2021.

[13] European Medicines Agency (EMA) . Medicines Sarclisa. 2022. Accessed February 28, 2023. https://www.ema.europa.eu/en/medicines/human/summaries‐opinion/sarclisa‐0

[14] Mikhael J , Richter J , Vij R , et al. A dose‐finding phase 2 study of single agent isatuximab (anti‐CD38 mAb) in relapsed/refractory multiple myeloma. Leukemia. 2020;34(12):3298‐3309. 32409691 10.1038/s41375-020-0857-2 PMC7685976

[15] Martin T , Strickland S , Glenn M , et al. Phase I trial of isatuximab monotherapy in the treatment of refractory multiple myeloma. Blood Cancer J. 2019;9(4):41. 30926770 10.1038/s41408-019-0198-4 PMC6440961

[16] Burova E , Hermann A , Waite J , et al. Characterization of the anti‐PD‐1 antibody REGN2810 and its antitumor activity in human PD‐1 Knock‐In mice. Mol Cancer Ther. 2017;16(5):861‐870. 28265006 10.1158/1535-7163.MCT-16-0665

[17] Lesokhin AM , Ansell SM , Armand P , et al. Nivolumab in patients with relapsed or refractory hematologic malignancy: preliminary results of a phase Ib study. J Clin Oncol. 2016;34(23):2698‐2704. 27269947 10.1200/JCO.2015.65.9789 PMC5019749

[18] Hallett WH , Jing W , Drobyski WR , Johnson BD . Immunosuppressive effects of multiple myeloma are overcome by PD‐L1 blockade. Biol Blood Marrow Transplant. 2011;17(8):1133‐1145. 21536144 10.1016/j.bbmt.2011.03.011

[19] Zhu C , Song Z , Wang A , et al. Isatuximab acts through fc‐dependent, independent, and direct pathways to kill multiple myeloma cells. Front Immunol. 2020;11:1771. 32922390 10.3389/fimmu.2020.01771 PMC7457083

[20] Chen L , Diao L , Yang Y , et al. CD38‐mediated immunosuppression as a mechanism of tumor cell escape from PD‐1/PD‐L1 blockade. Cancer Discov. 2018;8(9):1156‐1175. 30012853 10.1158/2159-8290.CD-17-1033 PMC6205194

[21] US Food and Drug Administration . FDA Alerts Healthcare Professionals and Oncology Clinical Investigators about two clinical trials on hold evaluating KEYTRUDA® (pembrolizumab) in patients with multiple myeloma. 2022; 2017.

[22] Kumar S , Paiva B , Anderson KC , et al. International Myeloma Working Group consensus criteria for response and minimal residual disease assessment in multiple myeloma. Lancet Oncol. 2016;17(8):e328‐e346. 27511158 10.1016/S1470-2045(16)30206-6

[23] Zucali PA , Lin CC , Carthon BC , et al. Targeting CD38 and PD‐1 with isatuximab plus cemiplimab in patients with advanced solid malignancies: results from a phase I/II open‐label, multicenter study. J Immunother Cancer. 2022;10(1):e003697. 35058326 10.1136/jitc-2021-003697 PMC8783811

[24] Pillai RN , Ramalingam SS , Thayu M , et al. Daratumumab plus atezolizumab in previously treated advanced or metastatic NSCLC: brief report on a randomized, open‐label, phase 1b/2 study (LUC2001 JNJ‐54767414). JTO Clin Res Rep. 2021;2(2):100104. 34589982 10.1016/j.jtocrr.2020.100104 PMC8474375

[25] Mateos MV , Blacklock H , Schjesvold F , et al. Pembrolizumab plus pomalidomide and dexamethasone for patients with relapsed or refractory multiple myeloma (KEYNOTE‐183): a randomised, open‐label, phase 3 trial. Lancet Haematol. 2019;6(9):e459‐e469. 31327687 10.1016/S2352-3026(19)30110-3

[26] Usmani SZ , Schjesvold F , Oriol A , et al. Pembrolizumab plus lenalidomide and dexamethasone for patients with treatment‐naive multiple myeloma (KEYNOTE‐185): a randomised, open‐label, phase 3 trial. Lancet Haematol. 2019;6(9):e448‐e458. 31327689 10.1016/S2352-3026(19)30109-7

[27] Dimopoulos M , Bringhen S , Anttila P , et al. Isatuximab as monotherapy and combined with dexamethasone in patients with relapsed/refractory multiple myeloma. Blood. 2021;137(9):1154‐1165. 33080623 10.1182/blood.2020008209 PMC7933767

[28] Koiwai K , El‐Cheikh R , Thai HT , et al. PK/PD modeling analysis for dosing regimen selection of isatuximab as single agent and in combination therapy in patients with multiple myeloma. CPT Pharmacometrics Syst Pharmacol. 2021;10(8):928‐940. 34185964 10.1002/psp4.12666 PMC8376141

[29] Sunami K , Suzuki K , Ri M , et al. Isatuximab monotherapy in relapsed/refractory multiple myeloma: a Japanese, multicenter, phase 1/2, safety and efficacy study. Cancer Sci. 2020;111(12):4526‐4539. 32975869 10.1111/cas.14657 PMC7734004

[30] Richardson PG , Perrot A , San‐Miguel J , et al. Isatuximab plus pomalidomide and low‐dose dexamethasone versus pomalidomide and low‐dose dexamethasone in patients with relapsed and refractory multiple myeloma (ICARIA‐MM): follow‐up analysis of a randomised, phase 3 study. Lancet Oncol. 2022;23(3):416‐427. 35151415 10.1016/S1470-2045(22)00019-5

[31] Mikhael J , Belhadj‐Merzoug K , Hulin C , et al. A phase 2 study of isatuximab monotherapy in patients with multiple myeloma who are refractory to daratumumab. Blood Cancer J. 2021;11(5):89. 33980831 10.1038/s41408-021-00478-4 PMC8116334

[32] Djebbari F , Poynton M , Sangha G , et al. Outcomes of anti‐CD38 isatuximab plus pomalidomide and dexamethasone in five relapsed myeloma patients with prior exposure to anti‐C38 daratumumab: case series. Hematology. 2022;27(1):204‐207. 35134321 10.1080/16078454.2022.2028978

