# Supplementary material for: Isatuximab in combination with cemiplimab in patients with relapsed/refractory multiple myeloma: A phase 1/2 study
Source: Cancer Med. 2023 Mar 3;12(9):10254–66. doi: 10.1002/cam4.5753 (PMC10225222; doi:10.1002/cam4.5753)

**SUPPLEMENTARY INFORMATION**

**Methods**

Cytogenetic analysis was performed on bone marrow collected at screening (baseline sample) by fluorescence *in situ* hybridization (FISH) to analyze 17p deletion, t(4;14), and t(14;16) abnormalities.

Immune cell populations, including CD3, CD4, and CD8 T cells, NK cell (CD45+CD3-CD56+/++) subsets, and CD38+ and PD-1+ (equivalent to CD279+) cells, were characterized by multiparametric flow cytometry analysis and results were reported as the percentage of cells on leukocytes or specific subsets. These analyses were performed on bone marrow aspirate and blood samples collected at screening and Day 1 of Cycle 3.

***Pharmacokinetic analysis***

Blood samples were collected at selected time points during Cycle 1 (predose, end of infusion [EOI], EOI+4h, start of infusion [SOI]+72h, SOI+168h) to perform Isa pharmacokinetics by non-compartmental analysis using Phoenix WinNonlin® version 8.2 (Pharsight). Isa (plasma) concentrations were measured using validated immunoassays with lower limit of quantitation of 5 µg/mL. Gyrolab Platform, a quantitative sandwich immunoassay using biotinylated anti-Isa antibodies bound by streptavidin beads within the Gyrolab Bioaffy CD microstructure for capture and Alexa Fluor® 647-conjugated CD38 antibody for detection, was used to measure functional Isa (Isa with ≥1 site available to bind target) plasma levels, with a lower limit of quantitation of 5.0 µg/mL and an upper limit of quantitation of 500 µg/mL.

***Immunophenotyping analysis***

For the participants with both screening (baseline) and Cycle 3 Day 1 samples available, approximately 20% of T cells (**Supplementary** **Figure 1A**) and 92% of NK cells (**Supplementary** **Figures 1B–C**) were CD38 positive in bone marrow aspirate (BMA) and blood at baseline, whereas approximately 10% and 20% of CD4+ T cells were PD-1 positive in BMA and blood, respectively (**Supplementary** **Figure 1D**). In both treatment arms (ie, Isa alone, Isa+Cemi), a decrease in total NK cells was observed at Day 1 of Cycle 3 in BMA (median decrease: 82% and 67%; n = 11 and 19, respectively) and blood (median decrease: 76% and 75%; n = 17 and 41, respectively) (**Supplementary** **Figure 1E**).

In BMA, a large decrease in CD38+ cells was observed among NK CD56+ bright cells (median decrease: 79% and 80%; n = 3 and 8, respectively) (**Supplementary** **Figure 1B**) and NK CD56+ dim cells (median decrease: 96% and 91%; n = 5 and 7, respectively) (**Supplementary** **Figure 1C**). This decrease was also observed among CD3 T cells (median decrease: 88% and 98%; n = 10 and 19, respectively) (**Supplementary** **Figure 1A**), CD4 T cells (median decrease: 92% and 96%; n = 10 and 9, respectively) (**Supplementary** **Figure 1F**), and CD8 T cells (median decrease: 88% and 96%; n = 10 and 14, respectively) (**Supplementary** **Figure 1G**). Similar findings were observed in blood (**Supplementary Figures 1A, 1B, 1C, 1F, and 1G**).

In BMA and blood for the Isa+Cemi arm, a decrease in PD-1+ CD4 T cells (median decrease: 81% and 97%; n = 9 and 16, respectively) was observed whereas an increase was seen in the Isa arm (median increase: 70% and 21%; n = 11 and 17, respectively) (**Supplementary** **Figure 1D**). In blood for the Isa+Cemi arm, a median decrease of 99% of PD-1+ CD8 T cells was observed (n = 18), whereas no decrease was observed for the Isa arm (n = 17) (**Supplementary** **Figure 1H**).

In both treatment arms (Isa alone, Isa+Cemi), a decrease of CD38 expression was observed in clonal plasma cells from BMA (median decrease in mean fluorescence intensity [MFI]: 54% and 58%; n = 9 and 10 for Isa alone and Isa+Cemi, respectively; data not shown). For the Isa arm, a median increase in MFI of 23% of PD-1+ expression was observed in clonal plasma cells from BMA, (n = 9), whereas no increase was observed in the Isa+Cemi arm (n = 10; data not shown).

At baseline, in the daratumumab-pretreated group compared with the daratumumab-naïve group, a slight trend was observed for a higher percentage of clonal plasma cells from BMA (median: 8.8% and 3.3%; n = 16 and 38) (**Supplementary** **Figure 2A**), a higher percentage of PD-1+ CD8 T cells in blood (median: 19.4% and 14.7%; n = 24 and 65) (**Supplementary** **Figure 2B**), and a higher percentage of Ki67+ CD8 T cells (median: 2.7% and 2%; n = 20 and 64) in blood (**Supplementary Figure 2C**). There was a slight trend for a lower percentage of CD38+ CD8 T cells in blood (median: 20.8% and 24.9%; n = 23 and 68) and BMA (median: 14.6% and 24.3%; n = 22 and 65) (**Supplementary** **Figure 2D**). Nevertheless, none of these trends were statistically significant.

On clonal plasma cells from baseline BMA, 29% less CD38 (median MFI: 9686 and 13718; data not shown) and 34% more PD-1 expression (median MFI: 1898 and 1413; data not shown) were observed in the daratumumab-pretreated group (n = 16) compared with the daratumumab-naïve group (n = 37). Similar baseline levels of NK cells were observed in blood (3%) and BMA (1.3%) in both daratumumab-naïve and -pretreated groups (**Supplementary** **Figure 2E**).

**Supplementary Tables**

**Supplementary Table 1.** Patient disposition (randomized population)

|  | Isa (n = 34) | Isa+CemiQ2W (n = 36) | Isa+CemiQ4W (n = 36) |
| --- | --- | --- | --- |
| **Randomized and not treated** | 0 | 1 (2.8) | 0 |
| **Randomized and treated** | 34 (100) | 35 (97.2) | 36 (100) |
| Patients still on treatment | 5 (14.7) | 5 (13.9) | 4 (11.1) |
| Patients with definitive discontinuation | 29 (85.3) | 30 (83.3) | 32 (88.9) |
| **Reason for definitive treatment discontinuation** |  |  |  |
| Adverse event | 0 | 1 (2.8) | 7 (19.4) |
| Progressive disease | 28 (82.4) | 26 (72.2) | 24 (66.7) |
| Withdrawal by subject | 1 (2.9) | 2 (5.6) | 0 |
| Other | 0 | 1 (2.8) | 1 (2.8) |
| **Reason for treatment withdrawal by subject** |  |  |  |
| Adverse event | 1 (2.9) | 1 (2.8) | 0 |
| Other | 0 | 1 (2.8) | 0 |

Percentages are calculated using the number of patients randomized as the denominator. Patients treated but not randomized are tabulated according to treatment actually received (as treated).

Definitive treatment discontinuation is defined as the discontinuation of all study drugs.

Cemi, cemiplimab; Isa, isatuximab; Q2W, every 2 weeks; Q4W, every 4 weeks

**Supplementary Table 2**. Response rates by prior daratumumab exposure (ITT/Randomized population)

|  | Isa (n = 34) | | Isa+CemiQ2W (n = 36) | | Isa+CemiQ4W (n = 36) | |
| --- | --- | --- | --- | --- | --- | --- |
|  | **No Dara (n = 23)** | **Dara  (n = 11)** | **No Dara**  **(n = 30)** | **Dara  (n = 6)** | **No Dara (n = 25)** | **Dara (n = 11)** |
| **Overall response** |  | |  | |  | |
| Responders (sCR, CR, VGPR, or PR) | 4 (17.4) | 0 | 9 (30.0) | 0 | 8 (32.0) | 0 |
| **Clinical benefit** |  | |  | |  | |
| Responders (MR or better) | 7 (30.4) | 1 (9.1) | 12 (40.0) | 1 (16.7) | 13 (52.0) | 1 (9.1) |

Cemi, cemiplimab; CI, confidence interval; CR, complete response; Dara, daratumumab; Isa, isatuximab; MR, minimal response; PR, partial response; Q2W, every 2 weeks; Q4W, every 4 weeks; sCR, stringent complete response; VGPR, very good partial response.

**Supplementary Table 3**. Overview of infusion reactions (All-treated/safety population)

|  | Isa (n = 33) | Isa+CemiQ2W (n = 37) | Isa+CemiQ4W (n = 35) |
| --- | --- | --- | --- |
| **Any IR** |  |  |  |
| All grades | 18 (54.5) | 15 (40.5) | 16 (45.7) |
| Grade 1 | 2 (6.1) | 0 | 2 (5.7) |
| Grade 2 | 16 (48.5) | 15 (40.5) | 14 (40.0) |
| **Action taken with Isa** |  |  |  |
| Dose not changed | 2 (6.1) | 0 | 2 (5.7) |
| Drug interrupted | 16 (48.5) | 15 (40.5) | 13 (37.1) |
| Drug withdrawn | 0 | 0 | 1 (2.9) |
| **Action taken with Cemi** |  |  |  |
| Dose not changed | 0 | 14 (37.8) | 14 (40.0) |
| Drug withdrawn | 0 | 0 | 1 (2.9) |
| Not applicable | 18 (54.5) | 1 (2.7) | 1 (2.9) |
| **Episodes experienced** |  |  |  |
| Only 1 | 14 (42.4) | 15 (40.5) | 15 (42.9) |
| ≥1 | 18 (54.5) | 15 (40.5) | 16 (45.7) |
| ≥2 | 4 (12.1) | 0 | 1 (2.9) |
| **Onset of IR** |  |  |  |
| First infusion | 18 (54.5) | 15 (40.5) | 16 (45.7) |
| Subsequent infusions | 0 | 0 | 0 |

Cemi, cemiplimab; IR, infusion reaction; Isa, isatuximab; Q2W, every 2 weeks; Q4W, every 4 weeks

**Supplementary Table 4**. Isatuximab pharmacokinetic parameters

| Mean ± SD  (Geometric mean) [CV%] | Single agent (n = 10) | Combination (n = 30) | Overall (n = 40) |
| --- | --- | --- | --- |
| C_max_ | 264 ± 68.0 | 247 ± 65.9 | 251 ± 66.0 |
| (μg/mL) | (256) [26] | (238) [27] | (243) [26] |
| t_max_* | 5.56 | 4.85 | 5.04 |
| (h) | (3.25 - 10.50) | (2.58 - 13.70) | (2.58 - 13.70) |
| AUC_1week_ | 23 000 ± 7250 | 21 100 ± 6450 | 21 600 ± 6620 |
| (μg•h/mL) | (22 000) [31] | (20 100) [31]^†^ | (20 600) [31]^‡^ |

Isatuximab maximum concentration was generally observed at the end of infusion.

AUC, area under the concentration-time curve; CV, coefficient of variation; Isa, isatuximab; PK, pharmacokinetics; SD, standard deviation

*Median (Min–Max)

^†^N = 29

^‡^N = 39

**Supplementary Figures**

**Supplementary Figure 1. Median relative changes from baseline to Cycle 3 Day 1 in (A) CD38+CD3+ T cells, (B) CD38+NK CD56bright cells, (C) CD38+NK CD56dim cells, (D) PD-1+CD4+ T cells, (E) NK cells, (F) CD38+CD4+ T cells, (G) CD38+CD8+ T cells, and (H) PD-1+CD8+ T cells.** Samples from bone marrow aspirate are shown on the top *of each panel and samples obtained from peripheral blood are shown on the bottom of each panel. Isa alone treatment group is shown on the left side of each panel and Isa+Cemi is shown on the right of each panel. PD-1+ cells are shown as CD279+ cells.


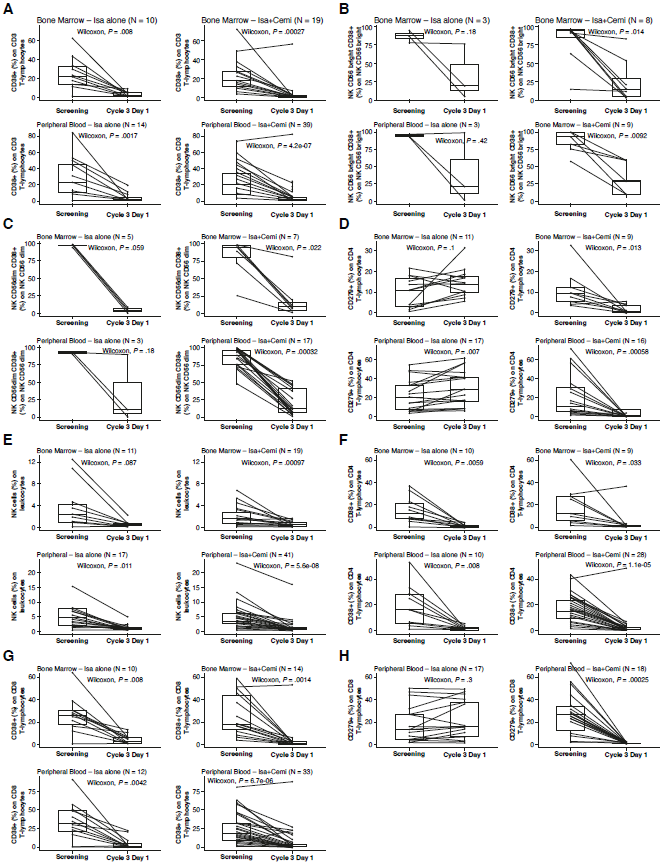


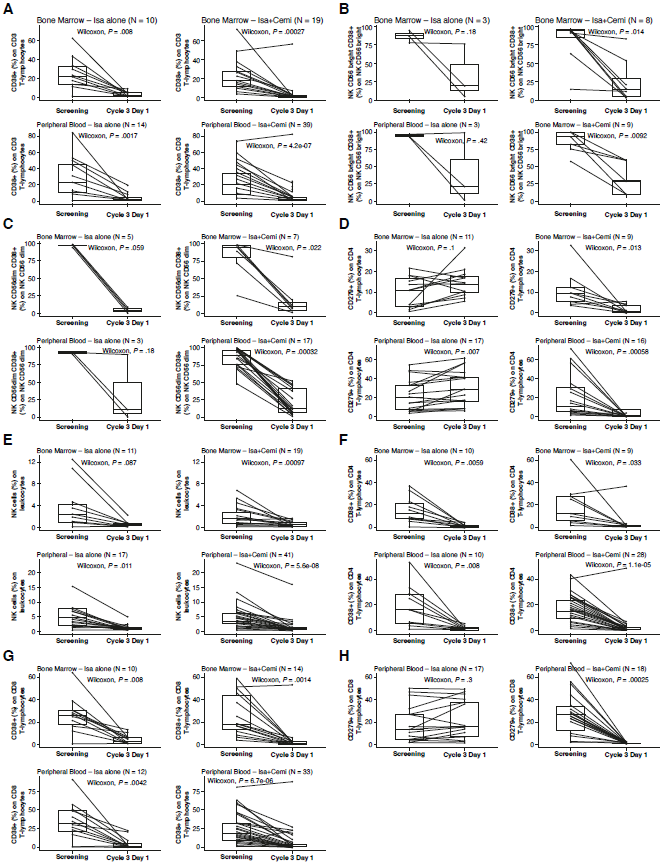


**Supplementary Figure 2. Median baseline levels of (A) clonal plasma cells, (B) PD-1+CD8+ T cells, (C) Ki67+CD8+ T cells, (D) CD38+CD8+ T cells, and (E) NK cells in daratumumab-pretreated and daratumumab-naïve patients.** Samples from bone marrow aspirate are shown on the left of each panel and samples obtained from peripheral blood are shown on the right of each panel. Dara, daratumumab. PD-1+ cells are shown as CD279+ cells.


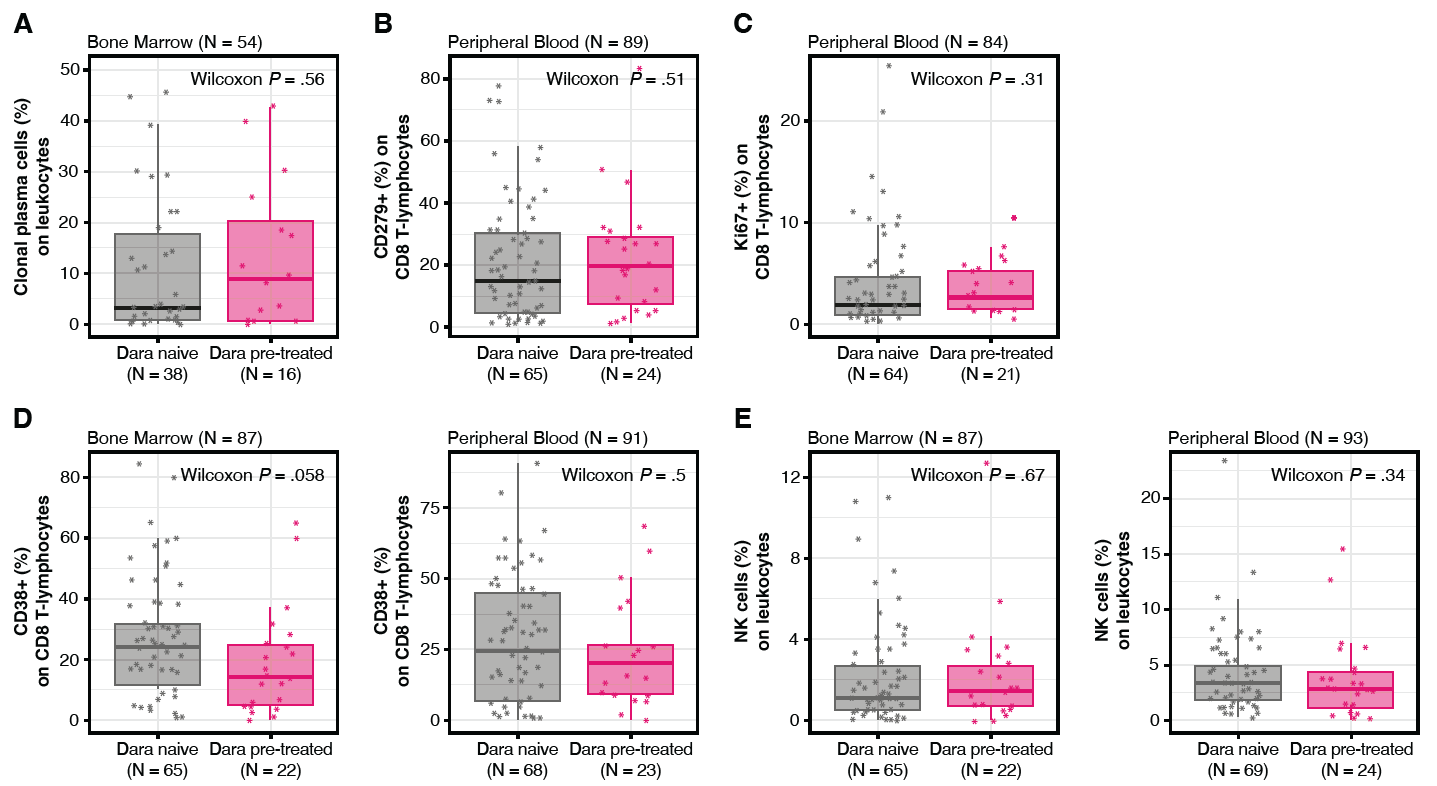

Supplement: Supplementary file 1 — Data S1. [file CAM4-12-10254-s001.docx]
